# Relationships between drinking habits, psychological resilience, and salivary cortisol responses on the Trier Social Stress Test-Online among Japanese people

**DOI:** 10.1186/s40359-023-01297-x

**Published:** 2023-08-28

**Authors:** Masaharu Ueno

**Affiliations:** Tobacco Academic Studies Center, 1-16-3, Yokokawa, Sumida-ku, Tokyo, 130-0003 Japan

**Keywords:** Alcohol, Cortisol, Online survey, Resilience, Trier Social Stress Test-Online

## Abstract

**Background:**

This study aimed to determine whether individual differences in resilience interacted with those in alcohol consumption habits in situations involving exposure to psychosocial stressors (Trier Social Stress Test-Online; TSST-OL). Additionally, we investigated whether individuals exhibiting resilience in their psychological scale scores showed biological responses that could be interpreted as resilience in stressful situations, such as the TSST-OL. We hypothesized that there would be no association between drinking habits and stress responses in the high-resilience group. Furthermore, high drinking habits would be associated with high stress responses in the low-resilience group.

**Methods:**

We recruited 22 and 20 individuals from the high and low-resilience groups, respectively, from among those who completed the online survey comprising the Alcohol Use Disorders Identification Test (AUDIT) and resilience scales; we excluded individuals with AUDIT scores of 15 or higher, and divided them by the median total resilience scale score. During the TSST-OL, self-rated stress measurement and saliva sample collection were performed seven times. Frozen samples were collected at the Tokyo site, and salivary hormonal (cortisol and dehydroepiandrosterone) levels were measured after transport in frozen state. Finally, 36 participants were included in the analysis of self-rated stress and cortisol levels.

**Results:**

We observed the typical subjective stress responses to the TSST-OL. People with higher psychological scale scores for resilience traits showed significantly higher salivary cortisol levels than those with lower scores. Due to deficiencies in the survey and experimental design, the classification criteria were changed and an exploratory analysis was performed to investigate the interaction of individual differences in resilience and drinking habits. In contrast to our expectation, those with low resilience scores showed stress responses, regardless of their drinking habits. Furthermore, those with high resilience and drinking habits showed a specific insensitivity to salivary cortisol levels. Their self-rated stress scores were similar to those of other groups.

**Conclusions:**

Our study showed the applicability of the TSST-OL in the Japanese population, the individual relationship between psychological resilience measures and biological stress responses, and a specific insensitivity in the salivary cortisol response as a result of individual differences in high resilience and drinking habits.

**Supplementary Information:**

The online version contains supplementary material available at 10.1186/s40359-023-01297-x.

## Background

Shikohin, a Japanese term related to luxury items such as alcohol, coffee, tea, and tobacco, contains nuances unique to Japan. We studied the positive role of shikohin in our lives and focused on the relationship between alcohol intake as a kind of shikohin and resilience. Alcohol intake may lead to alcohol-related problems. Thus, we included a screening test for alcohol to distinguish between consumption and problematic drinking. Studies suggest that rats with high alcohol consumption exhibit lower fear responses [1], which could reflect stress resistance—an aspect of resilience [2, 3]. In studies on humans, resilience has been strongly associated with a reduction in the risk of alcohol use disorders [4] and has been reported to moderate the relationship between stress and alcohol-related consequences [5]. Resilience is a phenomenon supported by multiple factors [6]. Researchers have argued that several aspects of resilience could be measured quantitatively using questionnaires and biomarkers. However, whether a psychologically resilient individual is biologically resilient remains unclear. Resilience in this study refers to the following two types: psychological resilience, indicated by psychological questionnaire scores and subjective stress reports, and biological resilience, defined by Yehuda et al. [3] as a specific pattern of hormonal variability as an acute stress response in laboratory experiments (i.e., high resistance or fast recovery). Many previous studies on resilience have focused primarily on people with or at risk of developing alcohol use disorders, and the relationship between resilience and drinking in light drinkers (those who enjoy drinking for pleasure) remains unclear.

We measured self-rated stress and salivary hormones, which included cortisol and dehydroepiandrosterone (DHEA), seven times during the experiment. These hormones were reported to be related to major depressive disorder and post-traumatic stress disorder as biomarkers of resilience. However, previous findings have been inconsistent [7]. An overview of several prior studies can provide insight into the relationships between cortisol variability, alcohol intake, and resilience. For example, a high cortisol response to acute stress has been suggested to modulate subjective responses to alcohol sedation in a dose-dependent manner [8]. Cortisol levels have also been shown to increase after alcohol administration in healthy men [9]. However, it has also been observed that alcohol consumption has no effect on the HPA axis in healthy light drinkers, suggesting that changes in the HPA axis response to alcohol may be more commonly found in higher-risk populations than in healthy drinkers [8]. Clay and Parker [10] showed that mild psychosocial stressors increased spontaneous alcohol consumption in healthy social drinkers and that heavy drinkers exhibited higher levels of subjective drinking enjoyment. Then, the cortisol response to acute stress and alcohol consumption could be not only simply correlated but also mediated by individual habit levels. Moreover, resilience has been reported to be strongly associated with a lower risk of developing alcohol use disorders [4] and to moderate the relationship between stress and alcohol [5]. Taken together, these prior findings suggest that stress exposure would elevate cortisol levels and promote alcohol consumption, but resilience could buffer the relationship between them. Resilient individuals would not demonstrate high alcohol consumption and alcohol-related problems. In contrast, low-resilience individuals would be at a higher risk of developing alcohol-related problems, and individuals with lower resilience and higher drinking habits would respond more markedly to acute stress, with higher cortisol elevations and slower recovery. To investigate these relationships, we designed a classification of groups based on resilience scores and drinking habits.

We focused on the possibility that the habit of consuming shikohin (e.g., alcohol) or having in such pleasures daily influences resilience. Thus, this study aimed to determine whether individual differences in resilience and alcohol consumption habits interact with each other. Furthermore, to understand the multiple aspects of resilience, we examined whether individuals determined as resilient by the psychological questionnaire show psychological and biological responses that could be interpreted as resilience to actual psychosocial stressors (Trier Social Stress Test-Online; TSST-OL [11]). In this study, two aspects of resilience—resistance and recovery [3]—were interpreted based on the degree of changes in the cortisol concentration from baseline to peak and from peak to recovery periods, respectively. This was followed by the TSST-OL experimental procedure described by Gunnar et al. [11], and included the baseline, peak, and recovery periods. We hypothesized that there would be no association between drinking habits and stress responses in the high-resilience group. Furthermore, high drinking habits would be associated with high stress responses in the low-resilience group. A high level of resilience was likely to indicate multiple factors that alleviate stress, and it would be unlikely for the stress response pattern to be influenced by alcohol consumption alone. Thus, the high-resilience group would exhibit smaller stress responses and have a more rapid recovery from increased stress responses at the baseline compared to the low-resilience group in TSST-OL. In contrast, the low-resilience group would be more likely to have problems related to alcohol consumption. Hence, the low-resilience group with high-frequency drinking habits would demonstrate higher stress responses and slower recovery at the baseline compared to the high-resilience group in the TSST-OL.

## Methods

### Deviation and changes from preregistration

The registration of this study design was opened before data collection at the Open Science Framework (10.17605/OSF.IO/CSJ57; date of registration: November 29, 2021, and updated: December 24, 2021).

However, it was not possible to conduct the process as outlined in the pre-registered framework, particularly the classification and analysis of groups. In the classification, we did not include a sufficient number of participants who exhibited low levels of drinking habits. Failure to classify the data according to the plan required a change in the analysis plan.

During the pre-registration, participants who answered “1: Monthly or less” to Q1 of the AUDIT Japanese version questionnaire were classified as the low-drinking group, those who answered “2: Two to four times a month” and “3: Two to three times a week” as the middle-drinking group, and those who answered “4: Four or more times a week” as the high-drinking group [12]. The three groups were analyzed along with the two groups of high and low resilience, for a total of six groups as follows: high-resilience–high-drinking group, high-resilience–middle-drinking group, high-resilience–low-drinking group, low-resilience–high-drinking group, low-resilience–middle-drinking group, and low-resilience–low-drinking group.

We conducted the initial survey, which included demographic items and exclusion criteria to reduce the participant load. Subsequently, those not excluded were requested to answer the resilience scale and Alcohol Use Disorders Identification Test (AUDIT) in a second survey. After the second survey, we excluded nondrinkers and problem drinkers (AUDIT score ≥ 15 points) and attempted to recruit those with high and low resilience scale scores (high-resilience group (n = 22); low-resilience group (n = 20)). Four individuals were excluded because they were unable to complete the experiment (high-resilience group (n = 21); low-resilience group (n = 17)). We then excluded two individuals whose cortisol levels were below the threshold (high-resilience group (n = 20); low-resilience group (n = 16)). We began the analysis when all data were available, at which point we realized that the classification criteria and selection process were inadequate as there was only one person in the low-resilience group. We realized that the drinking habit items from the AUDIT should have been considered, and the recruitment should have been adjusted to include a certain number of low-level drinkers. In addition, the AUDIT should have been included in the initial survey, considering the adjustment load. To reduce experimenter bias, the experimenter did not analyze the survey results prior to the experiment, which also contributed to the problem.

We classified the participants into two groups based on the median total score on the resilience scale—high-resilience and low-resilience groups. However, we changed another classification criteria for drinking habits and categorized the participants who responded to Q1 in the AUDIT with “1: Monthly once or less,” “2: Two to four times a month,” and “3: Two to three times a week” as the low-drinking group, and those who answered “4: Four or more times a week” as the high-drinking group. We found that there were 10 low drinkers and 6 high drinkers in the low-resilience group. In the high-resilience group, 7 were low drinkers and 13 were high drinkers. In light of this, the following four groups were created: low-resilience–low-alcohol (LRLA, n = 10), low-resilience–high-alcohol (LRHA, n = 6), high-resilience–low-alcohol (HRLA, n = 7), and high-resilience–high-alcohol (RHHA, n = 13). Data were analyzed using two-way repeated measures analysis of variance (ANOVA) with four groups and seven time points.

### Online survey

We recruited participants via an Internet research company (Macromill, Inc., Tokyo, Japan). The online survey was conducted in two stages, a screening survey and a main survey. Those who completed the two surveys were rewarded with cashable coupons according to the regulations of Macromill, Inc.

In the screening survey, participants responded to demographic items and the following items related to the inclusion criteria. The target population was “people who consumed alcohol on a daily basis,” “aged 20–69 years,” “males,” “did not suffer from any physical or mental illness at the time of the survey,” “people who owned a freezer,” “people who read the explanation of the online experiment and agreed to participate,” “could use a computer to participate in the experiment,” “lived in Tokyo, Saitama, Chiba, or Kanagawa prefectures,” “were able to come to Shinjuku station from their homes within 1.5 hours,” and “were able to talk online for more than three hours in a quiet environment using their home PC.” Individuals who did not meet these conditions were excluded. In addition, for the analysis of salivary hormones, we excluded people who had bleeding from the mouth in daily life due to stomatitis or gingivitis.

The main survey used five scales—the Japanese version of the AUDIT (10 items) to measure drinking habits and problem drinking [12] and four scales to measure cognition and utilization of resources related to resilience [13], which included cognition (20 items, ω = 0.960) and utilization (29 items, ω = 0.977) of intrapersonal resources as well as cognition (20 items, ω = 0.994) and utilization (30 items, ω = 0.992) of environmental resources. We used all factors in the four resilience scales and modulated three items from the phrase “at school” to “at school or work,” as it did not match the profile of the study participants. Participants were required to select the degree of their own cognition or behavior in stressful situations on four scales of resilience. Each item was assessed on a 5-point Likert scale (which ranged from 1 [very strongly disagree] to 5 [very strongly agree]).

The Japanese version of the AUDIT consisted of 10 items, and participants were asked to choose one of three items for Q9 and Q10 and one of five items for the other eight items. As in previous studies [12, 14], each item was scored from 0 to 4 points, and the total score of the 10 items was used to determine the degree of problematic drinking. We used the core-ten items from the Japanese version of AUDIT (Core AUDIT) and modified three items (Q 2, 9, and 10). In Q2, we changed the description in accordance with the methodology currently used to screen for alcohol-related problems in Japan. For Q9 and Q10, the number of choices changed from “0. 2. 4.” to “1. 2. 3” as per an online survey.

The survey respondents were classified into groups based on their scores and recruited to participate in the experiment by the research company staff. Participants with a score of 15 points or more in the Japanese version of the AUDIT were excluded as they were likely to be alcoholics. Furthermore, the research company staff excluded people who clearly had a problem in articulation or based on the content of their speech during the recruiting call. They also excluded those who responded poorly based on their response tendency, such as, for example, respondents who selected “1” for all the questions.

### Online experiment (TSST-OL)

A total of 1433 people responded to the online survey. From these, we first excluded nondrinkers and problem drinkers (AUDIT scores ≥ 15). Next, we calculated the total resilience scale scores and classified the participants into high (n = 603) or low (n = 604) groups based on their median scores. Participants were recruited by the staff of the research company (Macromill, Inc.) through recruiting calls in the order of highest and lowest scores. During the recruiting calls, the calls were evenly distributed to avoid age bias, and those who had problems answering the call were excluded. Calls were terminated when the number of participants in each age group was reached (high-resilience group: 4 participants in their 20s, 4 in their 30s, 5 in their 40s, 5 in their 50s, and 4 in their 60s; low-resilience group: 4 in their 20s, 4 in their 30s, 4 in their 40s, 4 in their 50s, and 4 in their 60s).

The TSST-OL was performed following the procedure described by Gunnar et al. [11]. Self-rated stress assessment and saliva collection were conducted at seven time points: (1) before the baseline period (after watching a calming video), (2) after the baseline period (before the TSST), (3) during stress (after speech preparation), (4) immediately after stress (after completion of the math task), (5) after stress (15 min after point 4), (6) during recovery period 1 (10 min after point 5), and (7) during recovery period 2 (10 min after point 6).

For self-rated stress, participants were asked to rate their stress level by responding to the item, “how stressed did you feel.” Each response was rated on a 5-point scale ranging from 1 = “not at all stressed” to 5 = “highly stressed.”

To measure the concentrations of cortisol and DHEA in the saliva, participants were asked to collect approximately 1.0 ml of saliva in microtubes (2 ml), which were frozen (approximately − 20℃) and then collected. We used a Cryovial (2 ml, Salimetrics, LLC, USA) and Saliva Collection Aid (SCA; Salimetrics, LLC, USA) for saliva collection. Hormonal assays in saliva were sourced from Yanaihara Institute, Inc. (Shizuoka, Japan). Cortisol (saliva) EIA Kit (Cat. No. YK241, assay range: 0.012-3.000 µg/dL) was used. The intra-assay and inter-assay coefficients of variation were 5.8% and 6.2%, respectively. The DHEA (Saliva) EIA Kit (Cat. No.YK290, assay range: 22.222–5400 pg/mL) was used. The intra-assay and inter-assay coefficients of variation were 7.8% and 8.7%, respectively.

**Fig. 1:**
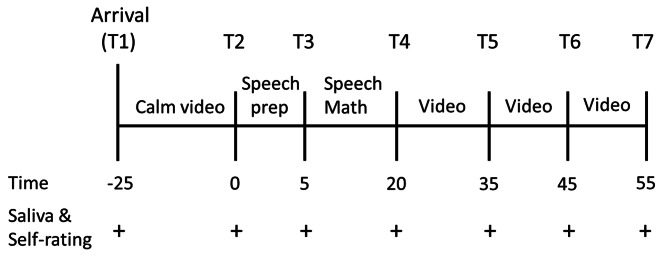
Overview of the TSST-OL procedure with the timing of saliva sampling and self-rated stress assessment. Speech prep = Speech preparation time

Prior to the TSST-OL, the experimenter and participant conducted a video call to check the communication condition via Zoom™ (https://zoom.us/). In addition, an explanation of the experiment, which included the restrictions (no eating, drinking, heavy exercise, smoking, and brushing teeth 1 h prior to the experiment, waking up before 9:00 am the day before and on the day of the experiment, and no alcohol consumption during those two days), and ethical considerations were discussed. Participants were mailed the saliva collection instruments (cryovial and SCA), a manual on saliva collection procedures, and the address of the sample collection site. On the day of the TSST-OL, the breakout and waiting room functions of Zoom™ were used to set up the experimenter and two evaluators in the main and breakout rooms, respectively. All three were not in the same room. There were always only two people in the main room—the experimenter and participant. Diurnal variations in cortisol are influenced by sleep and are the highest upon waking [15]. Considering this effect, participants were instructed to wake up at 9:00 am the day before and the day of the experiment. The experiments ran between 15:00 and 20:00 to control for diurnal rhythms in cortisol activity, which was consistent with previous studies of TSST-OL [11, 16]. Figure 1 shows an outline of the TSST-OL procedure.

After the participant entered the room, the experimenter informed them that their participation in the experiment could be interrupted at any time, and asked if they complied with the restrictions. The experimenter explained how to collect the saliva (pooling saliva in the mouth for 1 min) and required the participant to collect it, as demonstrated. After saliva collection was completed, the participant was asked to respond to the question on the self-rating of stress by pasting a URL via the chat function (T1).

Next, the participant was asked to watch a soundless video that showed a natural scene using the screen-sharing function. Simultaneously, the experimenter turned off the microphone and camera. The experimenter instructed the participants to relax and watch the video, but not to fall asleep. After 25 min, saliva samples and self-rated stress reports were requested (T2).

Subsequently, participants were led to the breakout room and were required to perform speech and math tasks. Two men assigned by ASMARQ Co., Ltd. participated as evaluators. They were trained by the experimenter to be expressionless and emotionless in their attitudes and behaviors prior to the TSST-OL. On the day of the experiment, they wore white lab coats and waited in the breakout room. The breakout room consisted of two evaluators and one participant. Participants were asked to prepare their speech immediately after they entered the room. The context for the prepared speech was that the participant had just started a new job and was asked by the supervisor to stand in front of 20 colleagues and introduce themselves. Participants were requested to give a good introduction to their own characteristics and talk about at least one strength and weakness. After these instructions, the evaluators turned off the video and measured the time for 5 min. At the end of min 4, one evaluator turned the camera off and announced the remaining time; after 5 min, saliva collection and self-rating stress report were requested (T3).

During the speech performance, participants were asked to stand up, set to the gallery mode in Zoom™, step back just enough to see their upper body and face, and give a 5-minute speech. The evaluator informed them that they were being recorded to evaluate their performance compared to others. If a participant was silent for more than 20 s, the evaluator asked the participant to continue the speech.

After the speech task was completed, a mathematical task was performed. Participants were instructed to keep subtracting 13 from 938. They were told that it was important to work accurately and quickly and that if they made a mistake, they would have to restart from the beginning. The two evaluators measured for 5 min with no facial expression. One evaluator checked whether the answers to the calculation were correct, pointed out any mistakes, and asked the participants to restart.

After the math task was completed, participants were led back to the main room, where the experimenter instructed them to perform saliva sampling and self-rating stress reporting (T4). The process was repeated for 15 min (T5), 25 min (T6), and 35 min (T7), after which the participants were asked to watch the nature video and report saliva collection and self-rating stress. In each case, the setting was similar to that of T2, and participants were instructed not to sleep. Finally, as a debriefing, the meaning of the TSST-OL was explained. Participants were informed of the point where the speech was not recorded, and were asked to freeze the saliva samples. The experimenter responded to any questions and comments. Only the main room was used during the experiment.

After the TSST-OL, participants brought their frozen saliva samples to the collection site in Tokyo. Samples were stored in a freezer (-20℃) for one day and then transported from the collection site to Yanaihara Institute Inc. at a frozen temperature (-80 ℃) by a professional transporter for biological samples (SAROUTE Co., Ltd.). The reason for involving these professionals was due to the COVID-19 situation in Japan from December 2021 to March 2022, when the study was conducted. Hence, intact saliva samples could not be transported by postal services or ordinary delivery companies without virus inactivation treatments.

Those who completed the experiment for approximately two hours were given rewards equivalent to JPY 16, 000. All participants completed the experiment.

### Participants and sample size rationale

We used G*Power 3.1.9.7 to estimate the required sample size for a two-way repeated measures ANOVA. We calculated the required sample size with “ANOVA: Repeated measures, within–between interaction” mode (Effect size f = 0.25, α = 0.05, 1 - β = 0.80, Correlation among repeated measures = 0.5, Nonsphericity correction ε = 1). We set the effect size to be moderate based on the pioneering study on TSST–OL [11]. As a result, 36 participants (number of groups = 6, number of measurements = 7) were set as the required and maximum sample size for statistical analyses.

As a result of the online survey, 42 Japanese male adults (aged 23–67 years, mean age = 45.14 years, SD = 13.06) participated. Of these, two (ID11 and 21) canceled their participation. Thus, there were 40 participants. Of these, two (ID7 and 23) were excluded as they were unable to perform the experimental task, and two more were excluded as their salivary cortisol concentrations were low and outside the measurement range. ID11 could not participate in the experiment because he was unable to communicate clearly with his face and voice when his Zoom^TM^ environment was checked prior to the experiment. ID21 canceled his participation in the experiment for personal reasons. ID7 became silent after about three minutes on both the speech and mental arithmetic tasks and did not respond when the evaluator asked him to continue. Similarly, ID23 was silent for five minutes during the speech task and did not respond to the evaluator’s requests to continue. Thus, ID7 and ID23 were excluded from the analysis because they were considered to have failed the TSST–OL. Finally, 36 participants (aged 23–67 years, mean age = 46.28 years, SD = 13.51) were included in the analysis of self-rated stress and salivary cortisol concentrations. Additionally, six individuals were excluded from the analysis of salivary DHEA concentrations due to low concentrations outside the measurement range. The results of the DHEA are provided as supplemental data (Supplementary Fig. 1). Our raw data, which included hormonal missing values, are available online (10.17605/OSF.IO/E9ZMH).

### Statistical analysis

Self-rating of stress and salivary hormone concentrations were analyzed using two-way repeated measures analysis of variance (ANOVA) with four groups and seven time points. Individual comparisons were evaluated using a simple main effect test and post-hoc Bonferroni test. The presence or absence of a significant difference was determined using the criterion of α = 0.05. We used the free statistical software js-STAR version 9.8.7j (https://www.kisnet.or.jp/nappa/software/star9/index.htm) for the ANOVA. The *ω* coefficients were calculated for each software using JASP version 0.16.3 [17].

## Results

A total of 36 individuals were included in the analysis of subjective stress and cortisol levels: the low-resilience–low-alcohol (LRLA, n = 10), low-resilience–high-alcohol (LRHA, n = 6), high-resilience–low-alcohol (HRLA, n = 7), and high-resilience–high-alcohol (RHHA, n = 13) groups. The ages of participants in the LRLA group were 23, 25, 28, 47, 48, 49, 51, 59, and 60 years; there were two 60 year-old participants (mean age = 45.00, *SD* = 14.47). Those in the LRHA group were aged 29, 37, 47, 59, 60, and 67 years (mean age = 49.83, *SD* = 14.77). Those in the HRLA group were aged 26, 27, 36, 46, and 53 years, with two participants aged 27 and two aged 53 years (mean age = 38.29, *SD* = 12.27). Those in the HRHA group were aged 27, 35, 36, 41, 44, 46, 58, 59, 60, 62, and 63 years, with two participants aged 58 and two aged 60 years (mean age = 49.92, *SD* = 12.26). A one-way ANOVA revealed no significant difference in age among the groups (*F* (3, 32) = 1.34, *p* = .28, *η*^2^ = 0.11).

### Self-rated stress

**Fig. 2:**
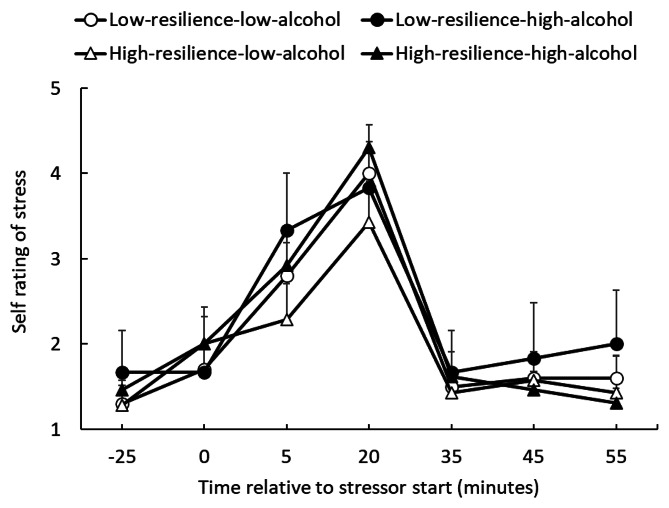
Self-rated stress assessment during TSST-OL sessions; participants were asked to rate their stress level. Each response was rated on a 5-point scale, which ranged from 1 =“not at all stressed” to 5 = “highly stressed.”

The degrees of self-rated stress in the four groups were analyzed using repeated measures ANOVA (total N = 36, Fig. 2). The main effect of the time point was significant (*F* (6, 192) = 37.00, *p* < .001, η_p_^2^ = 0.536). Multiple comparisons showed significantly higher values at T4 compared to all other time points (*p* < .05). The main effects of group (*F* (3, 32) = 0.46, *p* = .712, η_p_^2^ = 0.041) and interaction (*F* (18, 192) = 0.63, *p* = .873, η_p_^2^ = 0.056) were not significant.

### Salivary cortisol levels

**Fig. 3:**
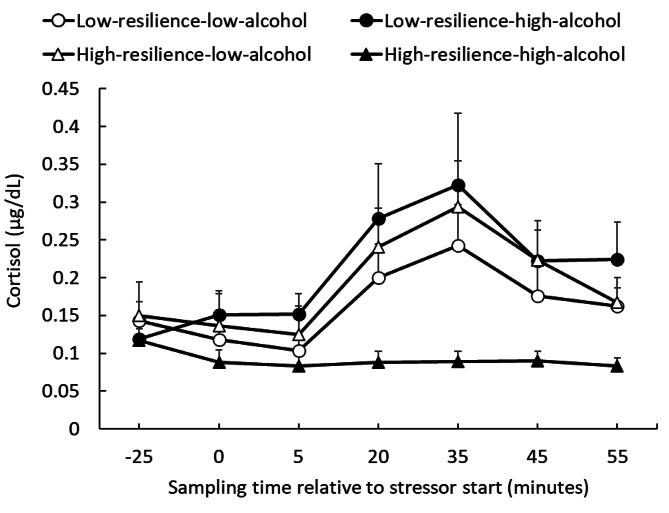
Salivary cortisol responses for TSST-OL in each group, notably, the low-resilient–low-alcohol (LRLA, n = 10), low-resilience–high-alcohol (LRHA, n = 6), high-resilience–low-alcohol (HRLA, n = 7), and high-resilience–high-alcohol (HRHA, n = 13) groups

Salivary cortisol levels in the four groups were analyzed using rmANOVA (total N = 36, Fig. 3). The main effects of group (*F*(3, 32) = 3.59, *p =* .02, η_p_^2^ = 0.252), time point (*F* (6, 192) = 14.05, *p* < .001, η_p_^2^ = 0.305) and interaction (*F* (18, 192) = 2.30, *p =* .003, η_p_^2^ = 0.178) were significant. The simple main effect test results showed significant group differences at T4, T5, T6, and T7 (*p* < .05). Multiple comparisons showed that the HRHA group had lower cortisol levels compared to the LRHA group at T4, T5, and T7. Furthermore, the HRHA group had lower cortisol levels compared to the HRLA group at T5 and T6. As shown in Fig. 3, the HRHA group had lower cortisol levels compared to the other groups. In addition, multiple comparisons showed that cortisol levels were significantly higher at T5 (peak) compared to at T2 (baseline) in the LRLA, LRHA, and HRLA groups (*p* < .05). As shown in Fig. 3, there was no significant increase in cortisol levels due to the TSST-OL in the HRHA group.

We counted responders and non-responders using the same procedures used in previous studies. People with increased cortisol levels of 0.054 µg/dL from baseline (T2) to peak (T5) were considered responders [11, 18]. As a result, six (60%) and four (40%) responders and non-responders were detected in the LRLA group, five (83%) and one (17%) in the LRHA group, four (57%) and three (43%) in the HRLA group, and one (8%) and 12 (92%) in the HRHA group, respectively. A chi-squared test of the responder rate revealed significant differences between the groups (χ^2^ (3) = 11.846, *p* = .008). Residual analysis showed that a significantly higher and lower percentage of responders and non-responders, respectively, emerged in the LRHA group (*p* < .05). The HRHA group had a significantly lower and higher percentage of responders (*p* < .01) and non-responders (*p* < .01), respectively. There was a significant difference in the cortisol response rate only in the two groups with high alcohol consumption habits.

## Discussion

This study aimed to determine whether individual differences in resilience were related to individual differences in drinking habits. We investigated whether the combination of high and low resilience scores and high and low drinking habits indicated a specific response pattern in stress situations. The hypotheses were that the high-resilience group would not interact with drinking habits, while the low-resilience group would exhibit different stress response patterns based on their drinking habits. The low-resilience–high-drinking group (LRHA group) would have a higher stress response compared to the other groups. A miscalculation in this study was the failure to collect a sufficient number of low drinkers to make a pre-designed classification. Therefore, the analysis was conducted with four groups instead of the six groups preliminarily assumed.

The results of cortisol responses showed that the HRHA group was markedly non-responsive. Regarding the percentage of responders and non-responders, the LRHA group showed a significantly higher responder rate compared to the other groups, whereas the HRHA group exhibited a significantly higher non-responder rate. This result suggested an interaction between daily drinking habits and resilience. There were no remarkable differences in salivary DHEA concentrations among the groups. Furthermore, this study aimed to determine whether psychologically resilient individuals were biologically resilient. As a result, resilience-related differences were not detected in hormonal measures during the experiment, despite the fact that the questionnaire survey measured resilience qualitatively before the experiment was conducted.

Resilience patterns (resistance or recovery), as suggested by Yehuda et al. [3], were not indicated by changes in subjective stress. There were also no differences in subjective stress levels between the two groups (HRLA and HRHA) that scored higher on the resilience questionnaire, that is, those with higher psychological resilience. In terms of the main effect of the groups, there were also no significant differences in both cortisol and DHEA levels. As Bonanno [19] summarized, regarding the relationship between resilience measured by questionnaires and cortisol as an acute stress response, several reports indicate that resilience scores did not show a direct association with HPA axis responses to laboratory stressors such as TSST [20–23]. The present results were consistent with these past studies.

The results of the chi-square test indicated that cortisol non-responders were exclusively concentrated in the HRHA group. A blunted cortisol response to acute stress has been indicated as a risk associated with psychiatric disorders [24, 25]. If the typical transition from baseline to peak shows an increase and then an overall value that is lower than the other groups, it might be interpreted as stress resistance by Yehuda et al. [3]. However, as the cortisol response in the HRHA group in this study did not increase at all, it would be more consistent with previous studies to consider it a risk factor rather than an expression of resilience. There were no notable differences in the resistance and recovery between the other groups. However, to examine recovery based on cortisol levels, the recovery periods may need to be longer, or a different definition of recovery should be applied [26]. As this study was the first to perform the TSST-OL in Japan, we followed a similar procedure to that used in a previous study [11]. Since our results showed that it was possible to perform the TSST-OL in Japan, the definition of recovery could be updated for a more precise analysis in future studies.

This study had some limitations. First, it was important that all the participants were habitual drinkers. In particular, the HA group in this study included those who drank “four or more times per week.” Hence, frequent daily alcohol consumption may have affected the hormonal dynamics, which included cortisol. The difference in response rates between the HA and LA groups also supported this assumption. In experiments, such as the TSST, which was designed for hormonal assays, alcohol consumption was usually restricted before the experiment [27], while drinking habits were not commonly used as a criterion for participant selection. Furthermore, this study excluded individuals with high levels of problematic drinking. Hence, the results could be different from those of typical studies on patients with alcohol use disorders and popular TSST studies. Second, regarding the overall percentage of cortisol responders, only 15 of 36 (42%) cortisol responders were detected. In previous studies on traditional TSST, the percentage of responders was > 70%, and even in TSST-OL, the occurrence rate was > 60% [11]. However, this percentage may vary depending on the sample of interest. For example, a pilot study in the adult version of the TSST-OL (TSST-OA) reported that 90% of the participants were female and the percentage of responders was 48% [28]. In contrast, Meier et al. [16] reported that participants comprised 55% female and 45% male, and the responder rate was 64% for the 1.5 nmol/L criterion [16]. The stress paradigm of our study did not robustly induce hormonal changes for several possible reasons—blood sugar levels, the speech scene setting, and the evaluator’s sex. Previous research indicated that blood sugar levels could influence cortisol reactivity [29]. Moreover, another study reported that glucose intake about an hour before TSST significantly increased cortisol responder rates, while fasting participants with lower blood glucose levels tended to be non-responders [30]. Therefore, the participants in this study were restricted from eating for one hour prior to the experiment for hormonal assays. As our experiment started at 15:00 or 18:00, some participants may have skipped lunch or not eaten anything since lunch. Thus, adjusting or modifying these restrictions may be necessary. As the first attempt to conduct an TSST–OL in Japan, we followed the pioneering study by Gunnar et al. [11] as much as possible and did not manipulate blood sugar levels (they did not manipulate blood sugar levels because of their focus on adolescents). In addition, a prior report demonstrated that glucose ingestion showed no significant effect on the responder rate in the TSST [31]. However, a recently published study in which glucose was ingested before stress even in the online version of the TSST for adults indicates that such a glucose treatment may be necessary in the future [16].

The theme of the speech task was also the same as that in Gunnar et al.’s [11] study, which involved introducing oneself as a newcomer and not self-presentation in a job interview, which was set in a typical TSST. This will need to be modified in future research.

Furthermore, the sex of the participants and evaluator in the TSST has been implicated in cortisol variability [32]. In this experiment, mainly because of resource limitations, the evaluators were both male, along with all the participants. Duchesne et al. [32] showed that in male participants, the baseline cortisol levels increased in cases involving two male evaluators, as compared to those with male and female evaluators. As the definition of the responders in this study was determined by the difference between baseline (T2) and peak (T5) cortisol levels, the higher baseline level may have affected the proportion of responders. In the future, we should consider using both male and female evaluators. Our results may differ from those of previous studies as only male habitual drinkers were included in the present study, given its objectives and limitations of resources. Third, this study did not examine women and/or non-drinkers. Previous research showed that sex was a crucial factor in the relationship between cortisol stress reactivity and psychiatric disorders [25]. Fourth, our setting of the inclusion/exclusion criteria is less rigorous compared to previous studies. The reason for this is that at the time of design, it was difficult to predict how many Japanese adult males, who were not specifically familiar with psychological experiments, would own a personal computer, be skilled in using a video calling application such as Zoom™, and be able to complete the stress test and saliva collection following the on-screen instructions. It was also highly possible that the collected data would be insufficient for analysis. We certainly did not apply strict controls for medication, premature birth, or serious medical history (e.g., cancer, organ transplant) at the time of the experiment, and this issue should be addressed in future studies.

Although this study focused on the positive aspects of drinking habits and investigated their association with stress responses, it is possible that drinking with others and drinking alone had different relationships with resilience and psychological well-being, as shown in a previous study [33]. In selecting participants, we were unable to identify under what situations they usually drank (with others or alone) and what psychological effects they experienced (positive or negative) as a result of drinking. This rigorous categorization and extraction of participant characteristics will be the focus of future research. Differences in cortisol response rates may be attributed to these participant characteristics. Alternatively, the online environment could be a factor that influenced the TSST results. Previous studies using the TSST-OL reported smaller responder rates compared to those using the traditional TSST (on-site) [11, 16]. Many participants in this experiment were also connected from their homes or other familiar environments, which would have allowed them to engage in the experiment under more relaxed conditions compared to those at a university or research institute.

## Conclusion

Despite the above mentioned limitations, we believe that the present study is novel. To the best of our knowledge, this is the first study using the TSST-OL to be conducted in Japan. It further demonstrates that the TSST-OL procedure proposed by Gunnar et al. [11] applies to Japanese adult male participants (aged 23–67 years). The results of the self-rated stress assessment detected typical subjective stress responses in the TSST, consistent with previous studies. This study provided evidence that the TSST experiment is applicable in Japan, even under the limitations of in-person experimentation due to the COVID-19 pandemic. We believe that the relationship between the frequent occurrence of non-responders in the HA group and vice versa in the LA group suggests an interaction between resilience and drinking habits. This is a novel finding that has not been observed in conventional studies, mainly those involving non-habitual drinkers. While drinking could certainly cause problems such as alcohol use disorders and addiction, it may also have certain beneficial roles, such as facilitating communication and enhancing positive emotions [34]. It would be desirable to describe both the positive and negative effects of shikohin [33] such as alcohol and coffee. We expect future development in these areas.

### Electronic supplementary material

Below is the link to the electronic supplementary material.

Supplementary Material 1

Supplementary Material 2

## Data Availability

Open data on psychological questionnaires, self-rated stress, and hormonal assays described in this article are available in the Open Science Framework (10.17605/OSF.IO/E9ZMH).

## Abbreviations

AUDIT: Alcohol Use Disorders Identification Test
DHEA: Dehydroepiandrosterone
HRHA: High-resilience–high-alcohol group
HRLA: High-resilience–low-alcohol group
LRHA: Low-resilience–high-alcohol group
LRLA: Low-resilience–low-alcohol group
TSST-OL: Trier Social Stress Test-Online
