# Supplementary material for: Relationships between drinking habits, psychological resilience, and salivary cortisol responses on the Trier Social Stress Test-Online among Japanese people
Source: BMC Psychol. 2023 Aug 28;11:250. doi: 10.1186/s40359-023-01297-x (PMC10464479; doi:10.1186/s40359-023-01297-x)
Supplement: Supplementary file 1 — Supplementary Material 1 [file 40359_2023_1297_MOESM1_ESM.docx]

**Supplementary Result (Salivary DHEA levels)**

Six patients with values below the detection range were excluded from this analysis. Hence, 30 patients were analyzed from the LRLA (n = 9), LRHA (n = 5), HRLA (n = 5), and HRHA (n = 11) groups.

Salivary DHEA levels in the four groups were analyzed using rmANOVA (total N = 30, Supplementary Fig. 1). The main effects of group (*F* (3, 26) = 1.45, *p =* .251, η_p_^2^ = .143) and interaction (*F* (18, 156) = 1.02, *p =* .441, η_p_^2^ = .105) were not significant. There was a significant main effect of the time point (*F* (6, 156) = 4.54, *p* < .001, η_p_^2^ = .149). Multiple comparisons showed that DHEA levels increased at T5 compared to those at T2 (*p* < .05), and values at T3 were lower compared to those at T1, T4, and T5 (*p* < .05). Supplementary Fig. 1 shows that DHEA decreased from the resting period (T1) to the stress period (T3) in the LRLA and HRLA groups, whereas no such tendency was observed in the LRHR and HRHA groups. In addition, DHEA increased from T2 to T5 in the LRHA group, but not in the HRHA group, which appeared to be flat. However, overall, there was no significant difference between the groups.


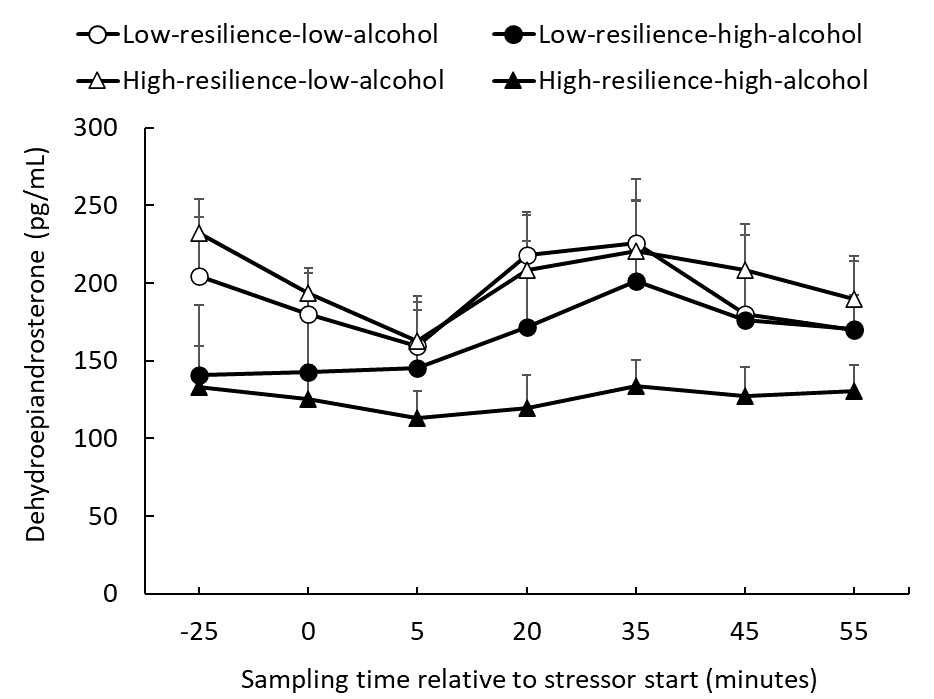


Supplementary Figure 1. Salivary DHEA responses for TSST-OL in each group.
