# Supplementary figures and images for: Relationships between drinking habits, psychological resilience, and salivary cortisol responses on the Trier Social Stress Test-Online among Japanese people
Source: BMC Psychol. 2023 Aug 28;11:250. doi: 10.1186/s40359-023-01297-x (PMC10464479; doi:10.1186/s40359-023-01297-x)

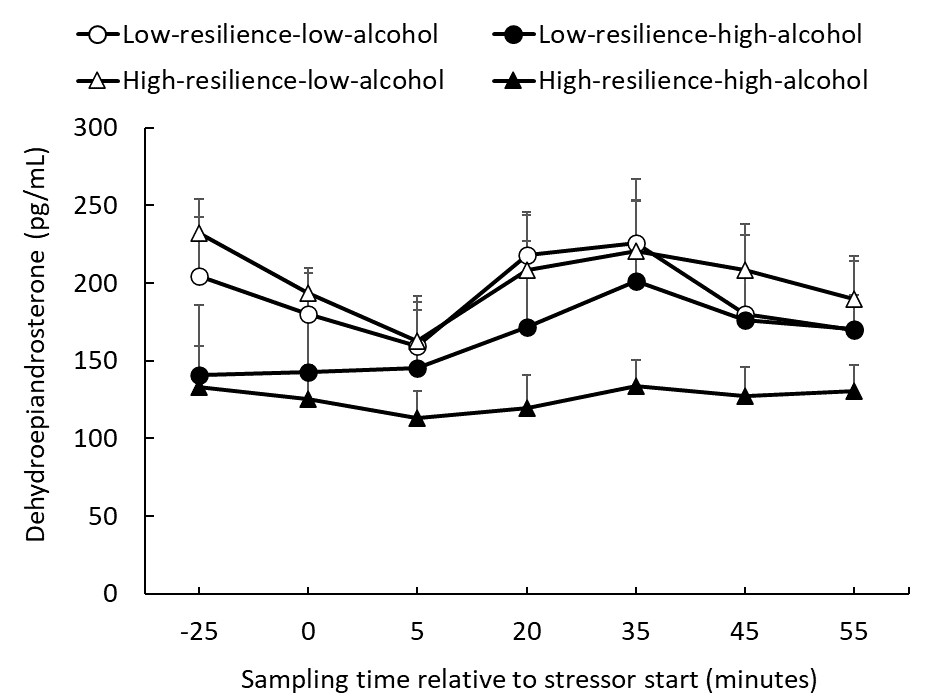

Supplement: Supplementary file 2 — Supplementary Material 2 [file 40359_2023_1297_MOESM2_ESM.jpg]
